# The nature of intraspecific and interspecific genome size variation in taxonomically complex eyebrights

**DOI:** 10.1093/aob/mcab102

**Published:** 2021-07-28

**Authors:** Hannes Becher, Robyn F Powell, Max R Brown, Chris Metherell, Jaume Pellicer, Ilia J Leitch, Alex D Twyford

**Affiliations:** 1 Institute of Evolutionary Biology, School of Biological Sciences, University of Edinburgh, Edinburgh, UK; 2 Royal Botanic Gardens, Kew, Richmond, Surrey, UK; 3 Wellcome Trust Genome Campus, Hinxton, Saffron Walden, UK; 4 Botanical Society of Britain and Ireland, Harpenden, Hertfordshire, UK; 5 Institut Botànic de Barcelona (IBB, CSIC-Ajuntament de Barcelona), Barcelona, Spain; 6 Royal Botanic Garden Edinburgh, Edinburgh, UK

**Keywords:** Genome size, polygenic trait, *Euphrasia*, ploidy, intraspecific variation, selection, pleiotropy, genomic repeats

## Abstract

**Background and aims:**

Genome size varies considerably across the diversity of plant life. Although genome size is, by definition, affected by genetic presence/absence variants, which are ubiquitous in population sequencing studies, genome size is often treated as an intrinsic property of a species. Here, we studied intra- and interspecific genome size variation in taxonomically complex British eyebrights (*Euphrasia*, Orobanchaceae). Our aim is to document genome size diversity and investigate underlying evolutionary processes shaping variation between individuals, populations and species.

**Methods:**

We generated genome size data for 192 individuals of diploid and tetraploid *Euphrasia* and analysed genome size variation in relation to ploidy, taxonomy, population affiliation and geography. We further compared the genomic repeat content of 30 samples.

**Key results:**

We found considerable intraspecific genome size variation, and observed isolation-by-distance for genome size in outcrossing diploids. Tetraploid *Euphrasia* showed contrasting patterns, with genome size increasing with latitude in outcrossing *Euphrasia arctica*, but with little genome size variation in the highly selfing *Euphrasia micrantha*. Interspecific differences in genome size and the genomic proportions of repeat sequences were small.

**Conclusions:**

We show the utility of treating genome size as the outcome of polygenic variation. Like other types of genetic variation, such as single nucleotide polymorphisms, genome size variation may be affected by ongoing hybridization and the extent of population subdivision. In addition to selection on associated traits, genome size is predicted to be affected indirectly by selection due to pleiotropy of the underlying presence/absence variants.

## INTRODUCTION

Genome size, defined as the amount of DNA in an individual’s unreplicated gametophytic nucleus (Greilhuber *et al.*, 2005), is associated with an organism’s life history, development, physiology, ecology, genome dynamics and evolution (Van’t Hof and Sparrow, 1963; Beaulieu *et al.*, 2008; Šímová and Herben, 2012; Greilhuber and Leitch, 2013; Bilinski *et al.*, 2018; Simonin and Roddy, 2018; Novák *et al.*, 2020; Roddy *et al.*, 2020). Genome size is estimated to show an ~64 000-fold variation across eukaryotes, and ~2440-fold variation in flowering plants (Pellicer *et al.*, 2018). Much is known about broad-scale variation in genome size across land plants and algae, with different phyla characterized by different genome size ranges (Pellicer and Leitch, 2020), and showing, in many cases, a strong phylogenetic signal (e.g. Weiss-Schneeweiss *et al.*, 2006; Vallès *et al.*, 2013; Wang *et al.*, 2016; Bainard *et al.*, 2019; Cacho *et al.*, 2021). Studies of diverse species differing in ploidy have shown that while whole genome duplication events initially lead to an increase in genome size, their subsequent evolution is often accompanied by genome downsizing over time (Leitch *et al.*, 2008; Leitch and Leitch, 2008; Pellicer *et al.*, 2010; Wong and Murray, 2012; Wendel, 2015; Zenil-Ferguson *et al.*, 2016; Wang *et al.*, 2021). Recently, community ecology studies have started to include data on genome size and to demonstrate its influence in shaping plant diversity (Guignard *et al.*, 2016, 2019).

While representative genome size estimates have been obtained for approximately two-thirds of flowering plant families (Pellicer and Leitch, 2020), variation between individuals and populations has typically received less attention, despite the increasing realization that such variation within species may be common (e.g. Šmarda *et al.*, 2010; Kolář *et al.*, 2017). Genome size has often been considered a property of a species, and there has been much debate as to whether it varies within species (Greilhuber, 2005; Gregory and Johnston, 2008; Šmarda and Bureš, 2010). Intraspecific differences in DNA content have been reported or are predicted between individuals with: (1) heteromorphic sex chromosomes (Costich *et al.*, 1991; Renner *et al.*, 2017), (2) different numbers of B chromosomes (Leitch *et al.*, 2007), dysploidy and aneuploidy, or (3) the presence/absence of specific DNA sequences. Such presence/absence variation may be subdivided into: (a) structural variants including insertion–deletion polymorphisms (indels), (b) copy number variation in protein-coding genes, commonly found in pan-genome studies (Hirsch *et al.*, 2014; W. Wang *et al.*, 2018; Gao *et al.*, 2019; Hübner *et al.*, 2019; Göktay *et al.*, 2021), and (c) copy number variation of rDNA copies (Long *et al.*, 2013) or of other genomic repeats (Chia *et al.*, 2012; Haberer *et al.*, 2020). Some differences, such as small indels, can be as small as one base pair, while others are large-scale (many megabases), including sequence duplications or loss of a dispensable chromosome. This presence/absence variation may be detectable by methods for estimating genome size, such as flow cytometry. Modern protocols using flow cytometry with appropriate reference standards, and following best practice approaches, can be accurate and highly precise (Greilhuber *et al.*, 2007; Pellicer *et al.*, 2021) and reveal genuine intraspecific variation. Consequently, there are an increasing number of well-documented reports of intraspecific genome size variation (e.g. Achigan-Dako *et al.*, 2008; Šmarda *et al.*, 2010; Díez *et al.*, 2013; Hanušová *et al.*, 2014; Blommaert, 2020).

**Fig. 1.**
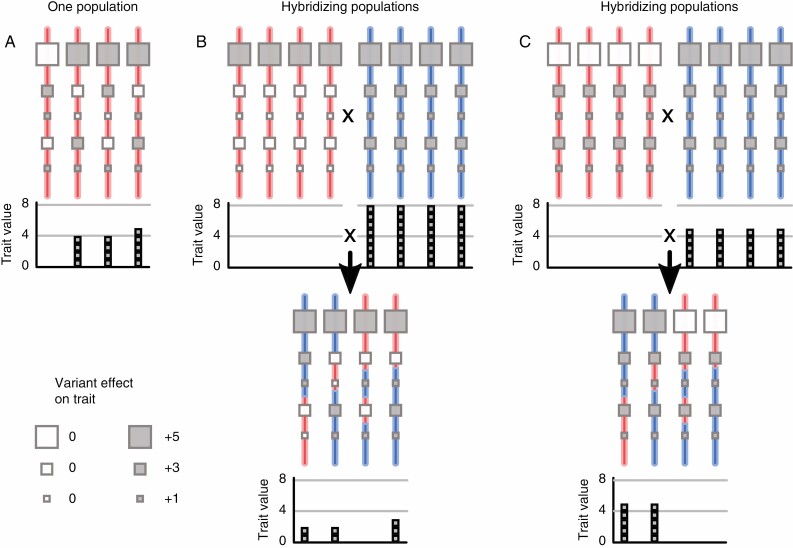
Schematic illustration of a polygenic trait, and its variability after hybridization. Each red or blue line represents an individual’s genome. Squares represent genetic variants with different effect sizes on a trait. The bar charts indicate individuals’ trait values, relative to the individual with the lowest value. (A) A population (or species) with genetic variability for the trait. The effect of hybridization between populations with different trait values depends on the genetic architecture of the trait difference. If the populations differ in many variants with small effects (B), recombinant offspring (denoted by mixed red and blue lines) are likely to have similar trait values. If, however, trait differences are due to a few variants with large effects (C), segregation in the recombinant offspring can produce higher trait variation. Applied to genome size, open squares correspond to DNA missing and filled squares to DNA present at some site in the genome, as detailed in the main text.

Our study considers genome size variation as polygenic, meaning heritable, and with a value affected by multiple independent loci in the genome (Fig. 1). Loci underpinning polygenic variation need not be protein-coding genes, but may also involve non-coding sequences including introns, promotors, trans elements or genomic repeats. Loci underpinning a polygenic trait may differ in their effect sizes, as shown by Koornneef *et al.* (1991) for flowering time in *Arabidopsis thaliana* (see also Napp-Zinn, 1955). Further, variants at a genetic locus are commonly pleiotropic, affecting multiple traits and thus potentially being the target of multiple selective effects. An early example of treating genome size as such is the study of Meagher *et al.* (2005) on the relationship between genome size and flower size in *Silene latifolia*, which showed correlations between floral traits and genome size in male plants of this dioecious species.

Here we explore genome size variation in British eyebrights (*Euphrasia* L., Orobanchaceae), a recently radiating taxonomically complex group. They comprise five diploid (2*n* = 2*x* = 22) and 15 tetraploid species (2*n* = 4*x* = 44) (Metherell and Rumsey, 2018). Recent genomic sequencing showed that British tetraploids are closely related allotetraploids, with one sub-genome derived from, or closely related to, British diploids (Becher *et al.*, 2020). The genus is an ideal group for investigating genome size variation within and between closely related species because species diversification is frequently postglacial (Gussarova *et al.*, 2008; X. Wang *et al.*, 2018), with many taxa being narrow endemics or recent hybrid species. *Euphrasia* therefore provides multiple opportunities to study genome size changes at the early stages of species divergence. Moreover, heterogeneous ecological conditions may promote local adaptation, and extensive hybridization may result in local geographical homogenization with variation in genome size structured by geography rather than by taxonomy, as seen previously in microsatellite and AFLP studies of population structure (Kolseth and Lönn, 2005; French *et al.*, 2008).

To investigate the nature of genome size variation in British *Euphrasia* species, we generated a comprehensive dataset of 192 genome size estimates across 13 species and ten hybrid combinations, supplemented with genomic sequence data to estimate the abundance of genomic repeats for 30 diverse diploids and tetraploids. Our study aims to answer the following questions: (1) How variable is genome size within species, between species and between ploidy levels? (2) What is the contribution of genomic repeats to genome size variation in British *Euphrasia*, and how does repeat content differ between the ploidy levels? (3) Does genome size variation correspond with known patterns of genetic structure and/or environmental variables in British *Euphrasia*? We discuss our results in the light of polygenic variation, and we argue for a closer integration of population genomics with research on genome size variation.

## METHODS

### The study system

**Fig. 2.**
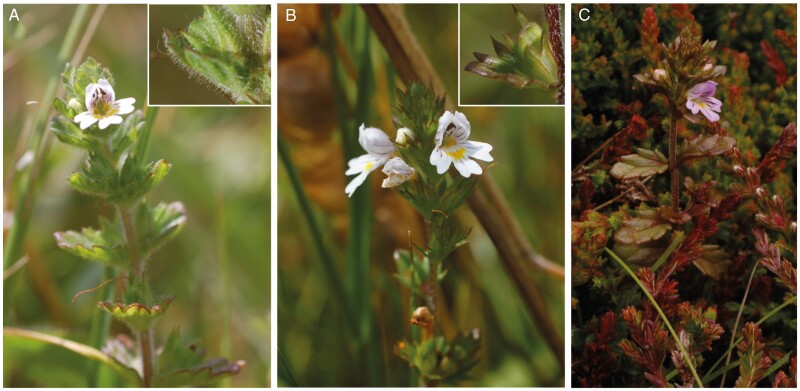
Morphological diversity in diploid and tetraploid British *Euphrasia*. (A) Diploid *Euphrasia rostkoviana* in South Wales. Inset shows long glandular hairs on seed capsule. (B) Tetraploid *Euphrasia arctica* in South Wales. Inset shows largely glabrous seed capsule. (C) Tetraploid *Euphrasia micrantha* in Shetland, Scotland.

British *Euphrasia* are a group of facultative hemiparasitic plants that are green and photosynthesize, but acquire up to 30 % of their carbon heterotrophically by parasitizing a range of different plant hosts (Těšitel *et al.*, 2010; Brown *et al.*, 2021). All British *Euphrasia* species are annuals. The diploid species group and the tetraploid group differ by a number of attributes (Fig. 2). The diploid species have long glandular hairs, bear generally large attractive flowers that are predominantly outcrossing (French *et al.*, 2005), and are largely restricted to England and Wales (Metherell and Rumsey, 2018). In contrast, tetraploid species are glabrous or possess short eglandular hairs, with smaller flowers that either self-fertilize or are mixed-mating, and are found throughout Britain. While many *Euphrasia* species are narrowly distributed, diploid *E. anglica* and *E. rostkoviana*, and a number of tetraploid species such as *E. arctica*, *E. confusa*, *E. micrantha* and *E. nemorosa* are particularly widespread in Britain. Hybridization is extremely common between species, and 71 hybrid combinations have been reported (Stace *et al.*, 2015). While hybridization between ploidy levels is suspected based on morphological intermediacy between four species combinations, only one confirmed naturally occurring triploid individual has ever been reported (Yeo, 1954), and attempts to generate interploidy hybrids via crossing have failed (Yeo, 1966). However, two diploid hybrid species with a mix of diploid and tetraploid characters are known, suggesting rare cross-ploidy hybridization may have important evolutionary outcomes (Yeo, 1956).

In terms of cytogenetic variation in British *Euphrasia*, we are not aware of reports of aneuploidy or B chromosomes, nor have these been documented in detailed cytogenetic work of European *E. rostkoviana* (Vitek and Kiehn, 1990). However, abnormal meiotic arrangements have been observed in diploid hybrids (Yeo, 1976; Vitek and Kiehn, 1990). There are previous genome size estimates for one species covered by this study, *E. rostkoviana*. The 1C-value of 2.73 pg for five samples from Bosnia and Herzegovina (Siljak-Yakovlev *et al.*, 2010) is considerably higher than our estimates reported below (see Results). However, *Euphrasia* tissue does not keep nor travel well, making flow cytometry challenging (Liebst and Schneller, 2005). Moreover, a wide range of ploidy levels are known in continental Europe (Gussarova *et al.*, 2008).

### Population and species-level genome size variation

#### Population sampling.

Our sampling for genome size estimation aimed to collect from across the diversity of British *Euphrasia* taxa, and from a wide geographical area. In total, 192 samples from 90 populations comprising 13 species and ten hybrid combinations were used for analysis, including extensive sampling of the widespread diploid *E. anglica* (23 individuals) and the widespread tetraploids *E. arctica* (43 individuals), *E. nemorosa* (22 individuals) and *E. micrantha* (17 individuals). Samples were either wild-collected on field trips to Wales, South-West England or Shetland (Scotland), and used directly for genome size estimates (54 samples) or contributed by botanical recorders from across Britain and Ireland as part of the Eye 4 Eyebrights public engagement project and grown from seed at the Royal Botanic Garden Edinburgh following Brown *et al.* (2020) prior to genome size estimation (138 samples). Our final dataset included most native species, except rare endemics of conservation concern such as *E. cambrica* and *E. rotundifolia*. A full list of samples analysed including their origin is given in Supplementary Data Table S1. The identification of species and hybrids was made by the *Euphrasia* taxonomic expert Chris Metherell, based on morphology.

#### Genome size measurements.

Nuclear DNA content of *Euphrasia* samples was estimated by flow cytometry using propidium iodide (PI)-stained nuclei, following the one-step method (see Pellicer *et al.*, 2021). Briefly, for each *Euphrasia* accession, two small leaves (~1–2 cm) were chopped together with the internal standard *Oryza sativa* ‘IR36’ (1C = 0.5 pg; Bennett & Smith, 1991) using a new razor blade, in a Petri dish containing 1 mL of ‘general purpose isolation buffer’ (GPB; Loureiro *et al.*, 2007), supplemented with 3 % PVP-40 and 0.4 μL β-mercaptoethanol. An additional 1 mL of buffer was added to the homogenate, and then this was filtered through a 30-μm nylon mesh to discard debris. Finally, the sample was stained with 100 μL of PI (1 mg/mL; Sigma) and incubated for 20 min on ice. For each accession analysed, one sample was prepared, and this was run three times on the flow cytometer. The nuclear DNA content of each sample run was estimated by recording at least 5000 particles (~1000 nuclei per fluorescence peak) using a Cyflow SL3 flow cytometer (Sysmex-Partec) fitted with a 100-mW green solid-state laser (Cobolt Samba). The resulting output histograms were analysed using the FlowMax software (v. 2.9, Sysmex-Partec) for statistical calculations. We report only genome size estimates for samples where the coefficients of variation (CV) of the sample and standard peaks in the flow histogram were less than 5 % (see Supplementary Data Fig. S1A and B for illustrative histograms of each ploidy level).

Where differences in genome size were detected within a species, combined samples containing at least two accessions were prepared following the same procedure as for individual runs. Genuine intraspecific variation was confirmed where multiple fluorescence peaks were identified from the combined run.

Throughout the paper we give 1C-values in pg; where necessary, published genome size values reported in Gbp were converted to pg using a conversion factor of 0.978 following Doležel *et al.* (2003).

### Repeat content variation

#### Sequence data generation.

We used a combination of existing and newly generated genomic sequencing data to investigate repeat variation in 31 samples comprising seven diploids and 23 tetraploids of *Euphrasia* plus *Bartsia alpina* as an outgroup. For existing genomic data, we downloaded short-read Illumina data from the Sequence Read Archive (SRA, see Supplementary Data Table S2). These included 18 samples in total, including 12 tetraploid samples from the isolated island of Fair Isle (Shetland, Scotland) generated for the study of Becher *et al.* (2020), which allowed us to study genomic repeat profiles in sympatric populations. This dataset also included a total of six representative diploid and tetraploid species from elsewhere in Britain.

We supplemented these previous data with newly generated sequence data from 11 additional UK samples representing a wider range of species and geographical locations, including 11 UK *Euphrasia* samples, an Austrian sample of *E. cuspidata* intended as a close outgroup to UK species, and *B. alpina* as an outgroup to the full sample set (Těšitel *et al.*, 2010; Scheunert *et al.*, 2012; A.D.T., unpubl. res.). Genomic DNA was extracted from 12 silica-dried samples and herbarium material of *E. cuspidata* using the Qiagen Plant Mini Kit (Qiagen), and used to prepare NEBUltra PCR-based libraries. Pooled libraries were sent to Edinburgh Genomics where they were run with other samples on a single lane of a HiSeq 2500 using high output mode with 125-bp paired-end sequencing.

#### Repeat content.

We ran the RepeatExplorer2 (RE) pipeline (https://repeatexplorer-elixir.cerit-sc.cz/Novák *et al.*, 2010, 2013, 2020) on a dataset of 25 000 randomly selected read pairs of each of the 31 samples (1 550 000 reads in total). This slightly exceeded the maximum number of reads that can be analysed with default settings (which depends on the data). Our dataset was therefore down-sampled to ~20 500 read pairs per sample. In comparative RE analyses, read numbers are often supplied in proportion to genome sizes to ensure repeats of similar genome proportion can be detected in all samples (Novák *et al.*, 2020). This logic does not apply here, where the British samples comprise 23 closely related tetraploids and six closely related diploids, with the diploid genome very similar to one of the tetraploid sub-genomes (Becher *et al.*, 2020). No matter what genome proportion is chosen per sample, there will always be more of the shared sub-genome than of the sub-genome restricted to tetraploids. To minimize mate overlaps of short insert sizes, each read was trimmed to 100 nucleotides. Further, we only used reads where at least 90 nucleotides had phred quality scores >30. To analyse the genomic repeat content, we excluded clusters annotated by RE as plastid DNA or Illumina process controls. Our numbers thus deviate slightly from RE’s automatic annotation.

#### Statistical analyses.

Most genome size analyses were conducted across all individuals or populations. However, for *E. arctica*, *E. anglica* and *E. micrantha*, where sampling covered most of their large geographical range in Britain, we also analysed data from each species separately. All analyses were done using R version 3.6.1 (R Core Team, 2019). For analyses of variance (ANOVAs) we used the function aov. To test whether sample means of genome size were significantly different, we used the function t.test, with Bonferroni correction in cases of multiple testing. To analyse how genome size variation was partitioned by ploidy, taxon and population we used ANOVA. To test the effect of ‘species’, we then re-ran the ANOVAs without hybrids (Table 1). To test the significance of genome size variance differences between species pairs, we divided the population mean genome sizes by each species’ grand mean (centring) and then applied an *F* test (R function var.test).

**Table 1.** Partitioning of genome size variation across *Euphrasia* species and hybrids

		d.f.	Sum Sq	Mean Sq	*F*	*P*
With hybrids	Ploidy	1	8.67	8.67	9505.96	**<2.0 × 10** ^**–16**^
	Taxon	21	0.11	0.01	6.00	**4.1 × 10** ^**–10**^
	Population	67	0.34	0.01	5.48	**7.8 × 10** ^**–15**^
	Residuals	102	0.09	0.00		
Without hybrids	Ploidy	1	7.96	7.96	8763.74	**<2.0 × 10** ^**–16**^
	Taxon	11	0.04	0.00	4.17	**6.9 × 10** ^**–5**^
	Population	62	0.33	0.01	5.92	**1.5 × 10** ^**–13**^
	Residuals	82	0.07	0.00		

The top analysis includes all 192 samples from 13 species and ten hybrids, and the lower analysis 157 samples comprising just the 13 species. Both ANOVA table detail the variance components (Sum Sq) accounted for by ploidy, taxon and population.

We tested the association between genome size and latitude using a mixed-effects model (R package nlme, function lme). For species analysed separately, we used linear models. We carried out Mantel tests to assess the relationship between geographical distance and genome size difference across all samples as done by Duchoslav *et al.* (2013). Unlike genetic data, which require population information, these Mantel tests could be carried out on individual-based genome size differences or population means. Isolation by distance was assessed using Mantel tests (R package vegan version 2.5-6) with 999 permutations.

To analyse genomic repeat patterns, we used hierarchical clustering and principal components analysis (PCA) on a matrix of the per-sample genome proportions of the 100 largest repeat clusters in R using the functions hclust and prcomp. Scaling the data (i.e. transforming per cluster the repeat frequencies so that their variance equals 1) leads to grouping of samples by dataset. For our final analyses, we omitted scaling, meaning that larger clusters contribute more to the overall variance as one would expect. *Bartsia alpina* was removed from the final PCA dataset, because its divergence from *Euphrasia* accounted for most of the variance in the data, obscuring variation within *Euphrasia*. To identify repeat clusters with large contributions to the first principal component, we selected those clusters which had absolute values >0.1 in the first eigenvector. We further used binomial-family generalized linear models to estimate the average genomic proportion individually for each repeat cluster. For each estimate, we computed the residual sum of squares as a measure of the variation in genomic abundance between individuals. We used linear models to assess the differences in relative abundance of individual repeat types between ploidy levels.

To investigate a possible association of individual repeat clusters with genome size, we used nine tetraploid samples for which we had both an estimate of the population average genome size and repeat data (samples marked with asterisks in Supplementary Data Table S2). We used the function cor.test to assess the significance level of any associations between the genome proportion of each individual repeat cluster and population average genome size.

### Data availability

The newly generated whole genome-sequencing data are available from the sequence read archive, Bioproject PRJNA678958. The genome size and repeat datasets and the scripts required to replicate our results are available on GitHub: https://github.com/hannesbecher/EuphrasiaGS).

## RESULTS

### Population and species-level genome size variation

Genome size estimates from all 192 individuals passed our quality checks. These samples came from 13 different species and ten hybrid combinations, including 40 diploid and 152 tetraploid individuals (Supplementary Data Table S1). Our samples covered a particularly wide geographical range for the large-flowered species *E. anglica* (diploid, sampling range 552 km) and *E. arctica* (tetraploid, 1152 km), and the small-flowered and highly selfing *E. micrantha* (tetraploid, 962 km).

The mean genome size across all tetraploids was 1.18 pg (s.e. 0.004 pg), which is 11 % less than twice the mean genome size of the diploids (0.66 pg, s.e. 0.008 pg). In the diploids, individual values ranged 1.2-fold, from 0.60 pg in *E. anglica* (population BED) to 0.73 pg in *E. anglica* in Dumfriesshire (E4E0085). In tetraploids there was a 1.3-fold variation, from 0.99 pg in *E. foulaensis* in Fair Isle (FIA105) to 1.33 pg in *E. arctica* in Orkney (E4E0033).

Intraspecific genome size ranges were widest in *E. arctica* (*n* = 43) and *E. foulaensis* (*n* = 13) (both 1.3-fold), and *E. anglica* (*n* = 23) (1.2-fold). *E. confusa* (*n* = 6), *E. nemorosa* (*n* = 22), *E. pseudokerneri* (*n* = 9) and *E. rostkoviana* (*n* = 9) had genome size ranges greater than 1.1-fold. While individuals with different genome size values were often found in distant populations, such as in *E. anglica* (0.60 and 0.73 pg, 525 km apart), and in *E. arctica* (1.04 and 1.33 pg, 903 km apart), we also found considerable genome size variation between populations in close proximity in *E. foulaensis* (0.99 and 1.25 pg, 2.5 km apart on Fair Isle) and in *E. confusa* (1.14 and 1.32 pg, same population). In all cases, tests to distinguish genuine intraspecific variation from technical artefacts confirmed the genome size differences reported between individuals (see Methods and Supplementary Data Fig. S1C and D). Generally, we found wider genome size ranges in taxa with more populations sampled. A notable exception was *E. micrantha* (genome size range 1.14–1.21 pg from 17 individuals analysed from nine populations, up to 962 km apart), which is discussed below.

**Fig. 3.**
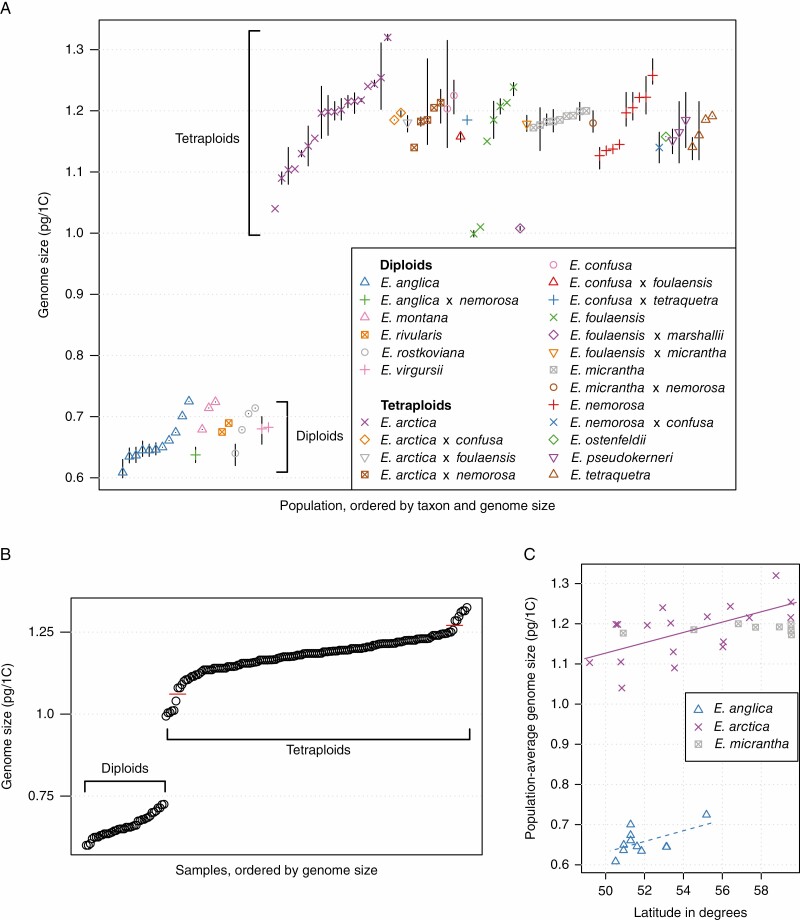
Patterns of genome size variation in British *Euphrasia*. (A) The distribution of population-average genome size for 90 populations of 23 taxa (13 species and ten hybrids). Vertical bars indicate the genome size range within each population where more than one individual was analysed. (B) Distribution of individual genome size estimates for all 192 samples. Horizontal red lines indicate the limits of the continuous part of the tetraploid genome size distribution. (C) Population-average genome sizes plotted against latitude for the three most widely sampled species. The solid purple line indicates a significant statistical relationship of genome size with latitude across 17 populations of *E. arctica*. This relationship was only marginally significant for 11 populations of *E. anglica* (dashed blue line). No significant association was found across nine populations of the highly selfing *E. micrantha*.

In ANOVAs, most of the overall genome size variation was explained by ‘ploidy’, while ‘taxon’ and ‘population’ accounted for smaller significant fractions (Table 1). ‘Population’ accounted for considerably more variation than ‘taxon’ – three or eight times, depending on whether hybrids were included in the analysis or not. This difference is due to the limited data available for most hybrids (Fig. 3A; Supplementary Data Table S1). The fact that ‘taxon’ generally accounts for only a small amount of the variance is reflected by the near-continuous distribution of genome sizes within each ploidy level (Fig. 3B). The distribution of tetraploid genome size values has two gaps, caused by a few exceptional individuals that are outliers in their genome size values. While most tetraploid genome size values are between 1.07 and 1.26 pg (red horizontal lines in Fig. 3B), six samples had lower (*E. arctica*, *E. foulaensis*, and *E. foulaensis* × *marshallii*), and seven higher, genome sizes (*E. arctica*).

Analyses of the three geographically widespread species with wider population sampling revealed that genome size variation was significantly partitioned by population for mixed-mating *E. anglica* (*F*_10,12_ = 9.86, *P* = 2.3 × 10^–4^) and *E. arctica* (*F*_17,25_ = 10.5, *P* < 1.7 × 10^–7^), but not for highly selfing *E. micrantha* (*F*_8,8_ = 0.31, *P* = 0.94). Furthermore, the variance in population average genome size was significantly lower in *E. micrantha* than in *E. anglica* (*F*_10,8_ = 11.65, *P* = 9.6 × 10^–4^) or *E. arctica* (*F*_17,8_ = 53. 2, *P* = 2.3 × 10^–6^).

Individual-based Mantel tests to link geographical distance and genome size variation were significant over all 40 diploid samples (Mantel statistic *r* = 0.25, *P* = 0.001) and all 152 tetraploids (*r* = 0.04, *P* = 0.01). We then carried out Mantel tests based on population averages to exclude the very local distance component. These tests were significant over all diploids (*r* = 0.27, *P* = 0.002) but not over all tetraploid populations (*r* = 0.04, *P* = 0.09). However, *E. arctica*, the most widespread tetraploid species, showed a pattern of isolation-by-distance at this level (*r* = 0.24, *P* = 0.015).

We confirmed a strong relationship between ploidy and latitude (ANOVA *F*_1,190_ = 18.79, *P* = 2.4 × 10^–5^), with diploids generally limited to lower latitudes (being particularly abundant in southern England, Supplementary Data Fig. S2) while tetraploids extend to the very north of Britain. However, there was no significant association between genome size and latitude within ploidy levels (treating taxon as a random effect, *t* = 0.63, *P* = 0.53). We then analysed the data for each of the three widely sampled species individually using linear models (Fig. 3C). There was a non-significant trend for the diploid *E. anglica* [slope = 0.013 pg/(degree latitude), *F*_1,9_ = 4.23, *P* = 0.07, *r*^2^ = 0.24]. Of the tetraploids, genome size increased significantly with latitude in *E. arctica* [slope = 0.013 pg/(degree latitude), *F*_1,16_ = 9.36, *P* = 0.008, *r*^2^ = 0.31], but not in *E. micrantha* (*F*_1,7_ = 0.34, *P* = 0.577).

### Variation in genomic repeat content

To investigate the nature of variants underpinning genome size variation, we analysed the genomic repeat content from whole genome sequencing data in 31 samples using the RE pipeline. RE’s output includes a set of annotated repeat clusters, representing individual repeat types. Our samples included *B. alpina* (Orobanchaceae), 29 British *Euphrasia* samples (six diploids and 23 tetraploids) and one Austrian diploid (Supplementary Data Table S2). Overall, 69.9 % of all *Euphrasia* reads analysed were identified as derived from repetitive DNA (i.e. they formed repeat clusters with genome proportions >0.01 %). The average genomic repeat contents of diploid and tetraploid *Euphrasia* samples differed, being 71.4 and 69.1 %, respectively (*F*_1,28_ = 8.14, *P* = 0.008). The repeat content for *B. alpina* was only 42.4 %, which is an under-estimate because repeats private to the species may have failed to form individual clusters given our sampling design and cut-off threshold.

The most abundant repeat family, ranging from 25 % in *E. anglica* (AN1) to 30 % in *E. cuspidata* (CU), was Angela, a type of Ty1/Copia long terminal repeat retrotransposon (LTR), which Wicker and Keller (2007) reported to range in length from 6.4 to 8.9 kb. Overall, all types of Ty1/Copia elements identified accounted for 30–39 % of each *Euphrasia* genome, while Ty3/Gypsy elements typically occupied just 3–6 % of the genome (Supplementary Data Table S2).

**Fig. 4.**
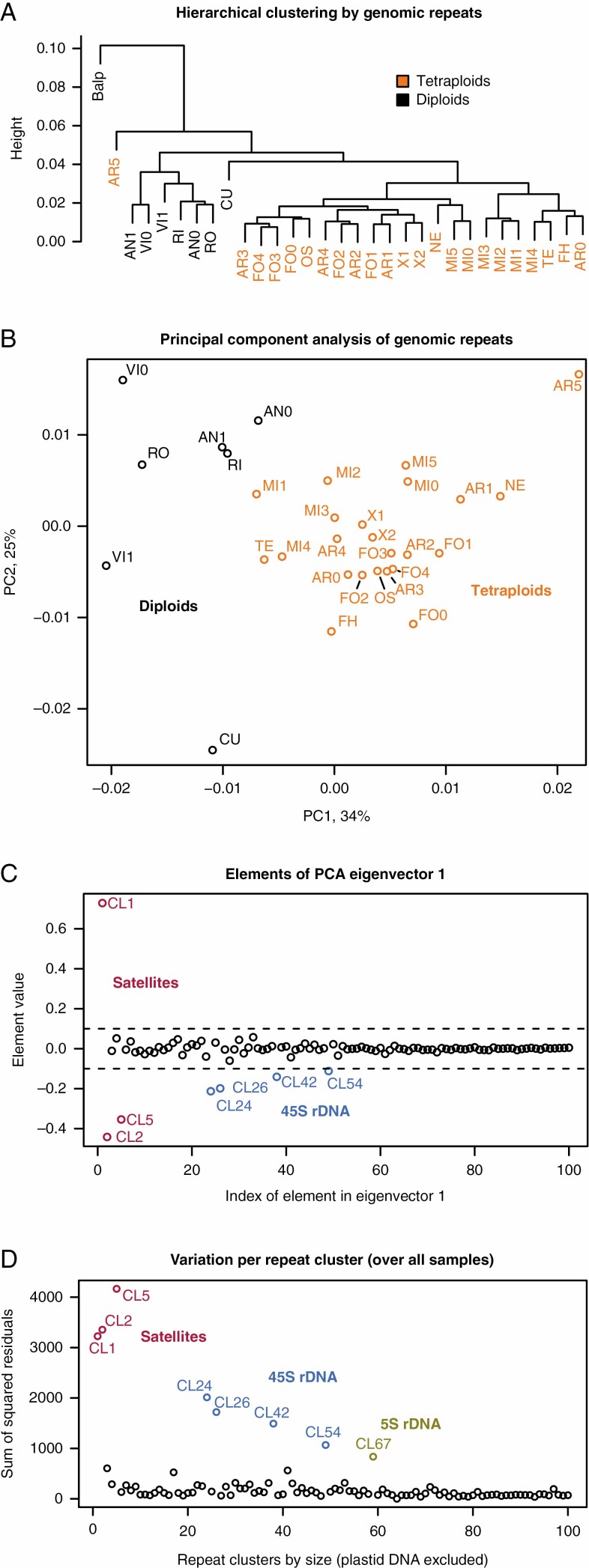
Clustering of *Euphrasia* samples based on genomic repeat content. (A) Hierarchical clustering shows grouping largely by ploidy. (B) A PCA of the relative proportions of the top 100 repeat clusters in 30 samples of *Euphrasia*. Diploids are shown in black and tetraploids in orange. (C) Contribution of each repeat cluster to the first principal component (of panel B). Clusters with negative values are enriched in diploids while those with positive values are enriched in tetraploids. (D) The extent of variation in the genomic proportions of all individuals for each repeat cluster. The abbreviations in A and B are: Balp, *Bartsia alpina* (outgroup), five diploid species (seven samples): AN, *E. anglica*; CU, *E. cuspidata*; VI, *E. vigursii*; RI, *E. rivularis*; RO, *E. rostkoviana*, seven tetraploid species and two tetraploid hybrids: AR, *E. arctica*; FO, *E. foulaensis*; MI, *E. micrantha*; NE, *E. nemorosa*; FH, *E. fharaidensis*; OS, *E. ostenfeldii*; TE, *E. tetraquetra*; and X, tetraploid hybrids.

To assess how genomic repeat profiles in samples from different populations correspond with species identity based on morphology, we used hierarchical clustering and PCA. We focused our analyses on the largest 100 repeat clusters, which together account for ~50 % of each genome in both diploids and tetraploids. Each smaller repeat cluster had a genomic proportion of <0.7 % in each sample. Hierarchical clustering resulted in a tree that grouped samples largely by ploidy, rather than species identity, except for (1) a sample of the Austrian alpine *E. cuspidata* (CU), a species considered diploid, which grouped as sister to the tetraploids, and (2) tetraploid *E. arctica* from Cornwall (AR5), which grouped as sister to all other *Euphrasia* samples (Fig. 4A). All species with multiple samples were mixed with other species in this tree. Among the sympatric samples from Fair Isle, *E. micrantha* (MI1-3) clustered separately from *E. arctica* (AR1-3) and *E. foulaensis* (FO1-4), both of which were mixed with other species, similar to previous patterns of clustering from single nucleotide polymorphism-based analyses (Becher *et al.*, 2020).

PCA without the outgroup *B. alpina* yielded a PC1 that explained 34 % of the variance in our repeat data, separating the diploid and tetraploid samples (Fig. 4B), whereas there was no clear separation by species. The samples for some species were spread widely across the plot [e.g. *E. arctica* (AR0-5) and *E. vigursii* (VI0, VI1)], while those of *E. micrantha* (MI0-5) grouped relatively tightly. Although this does not preclude the possibility of species-specific repeat patterns in *Euphrasia*, there are no major differences in the relative abundance of the common repeat types between the species. Within the 138 largest repeat clusters, none was species-specific (i.e. present in individuals of only one species). Within the largest 701 clusters, none was diagnostic for a species (i.e. none was present in all samples of one species but absent in all other samples; see also Supplementary Data Fig. S3).

To further analyse which repeat clusters separate diploids and tetraploids in the PCA (Fig. 4B), we plotted the elements of eigenvector 1, which correspond to the effect of each repeat cluster on the position of a sample along PC1 (Fig. 4C). Seven repeat clusters have a large effect on PC1, the satellite clusters CL1, CL2 and CL5, and all clusters of the 45S rDNA (CL24, CL26, CL42, and CL56). Satellite clusters CL1 and CL2 have monomer size peaks of ~145 nucleotides as commonly seen in centromeric repeats. In addition, some reads of CL1 and CL2 had paired-end mates in CL22, indicating physical proximity of the repeats within the genome. CL22, in turn, had been annotated as CRM, which is a type of Ty3/Gypsy chromovirus retrotransposon that commonly targets centromeric sequences (Nagaki *et al.*, 2003; Neumann *et al.*, 2011).

Among all 17 broad repeat types identified by RE (see Supplementary Data Table S2), we found significant differences between ploidy levels for two. Diploid genomes contained higher average proportions of 45S rDNA (4.9 %) than tetraploids (2.0 %, *F*_1, 28_ = 20.4, *P*_corr_ < 0.001), with the genomic proportion ranging from 1.7 to 5.7 % in diploids and from 0.8 to 3.4 % in tetraploids. Tetraploids contained, on average, more Ty1/Copia Ale elements (0.15 %) than diploids (0.09 %, *F*_1,28_ = 11.18, *P*_corr_ = 0.018). While our PCA approach had identified some satellites as highly differentiated in copy number (see above), differences over all satellites were not significant. This is because there was differential enrichment in the ploidy levels for CL1 vs. CL2 and CL5 (Fig. 4C). Overall, there is little differentiation in genomic repeats between the ploidy levels except for tandem repeats.

We also assessed the variation in repeat content over all samples for each repeat cluster. The eight most variable clusters (i.e. having the biggest differences in repeat proportions between individuals, Fig. 4D), are all tandem repeats (satellites including rDNA). The first seven are the same repeats that separated the ploidy levels in the PCA. The eighth most variable repeat (CL67), which is variable in both ploidy levels, corresponds to the 5S rDNA.

Of the samples analysed with RE, nine tetraploids were from populations which also had genome size estimates obtained in this study. Testing the largest 100, 200 and 1000 repeat clusters for correlations between genome size and abundance of individual repeat clusters, and correcting for multiple testing by Bonferroni correction, no repeat cluster showed a significant correlation between its abundance in an individual and the population-average genome size. All evidence from repetitive elements suggests that the genome size differences between *Euphrasia* individuals of the same ploidy levels are not due to large changes in the genomic proportion of any one specific repeat.

## DISCUSSION

In this study, we investigated the nature of genome size variation across taxonomically complex diploid and tetraploid British *Euphrasia*. We complemented a population survey of genome size variation with an analysis of genomic repeat composition from seven diploids and 23 tetraploid *Euphrasia*. Overall, we find notable genuine genome size variation of up to 1.3-fold between individuals of the same species. These values are comparable with reports for species such as *Dasypyrum villosum* (1.07-fold, Greilhuber, 2005), tetraploid *Festuca pallens* (~1.2-fold, Šmarda *et al.*, 2010), and *Sinningia speciosa* (1.25-fold, Zaitlin and Pierce, 2010). Within ploidy levels, we observed a continuum of genome size variation, though ploidy levels have discrete genome size ranges. Our study includes one interploidy hybrid, *E. anglica* × *E. confusa*, which was of diploid-level genome size, in accordance with the suggestion by Yeo (1956) that interploidy hybridization in British *Euphrasia* would give rise to diploids. Genome size differences within and between ploidy levels are not attributable to large copy number changes of individual DNA repeats, but rather to multiple presence/absence variants. Here, we first discuss the link between genome size variation and population dynamics/speciation history, highlighting how genome size variation is shaped by many similar processes as population-level sequence variation. We then consider the landscape of repeat dynamics and the potential association with *Euphrasia* polyploid genome history. Finally, we consider the wider implications of framing genome size variation in a population genetic framework.

### Genome size variation mirrors population genetic patterns

Population analyses have shown most genetic variation is not partitioned by *Euphrasia* species (Kolseth and Lönn, 2005; French *et al.*, 2008; Becher *et al.*, 2020), with only certain taxa, such as the moorland selfing species *E. micrantha*, being genetically distinct. For example, larger flowered mixed-mating species such as *E. arctica*, *E. confusa* and *E. nemorosa* lack genomic differentiation, and genetic structure corresponds to geography (French *et al.*, 2008). Here, we find genome size variation mirrors these findings of a lack of species divergence inferred from molecular data. Our results show *Euphrasia* taxa do not clearly show distinct genome size ranges possibly indicative of reproductive isolation, and instead show evidence of local hybridization leading to geographical differentiation (see below). Future taxonomic work will reappraise species boundaries using the joint evidence from morphological differentiation present in the field and plants grown in a common garden, and from patterns of genomic differentiation and genome size variation.

The continuous genome size distribution within ploidy levels, irrespective of species boundaries, resembles the findings of Hanušová *et al.* (2014) for species of the lycophyte *Diphasiastrum* at allopatric and sympatric sites. These authors concluded that considerable genome size variation within species resulted from introgression from other sympatric species. Depending on the sizes and number of segregating presence/absence variants (see schematic in Fig. 1B and C), hybridization between divergent populations may homogenize local genome sizes or introduce genome size differences. In our study, three populations from Fair Isle (one *E. foulaensis* × *E. marshallii* and two *E. foulaensis*) located within 5 km of each other show probable signals of introgression of presence/absence variations. These taxa show striking morphological differentiation, *E. foulaensis* × *E. marshallii* having a long hoary indumentum while *E. foulaensis* is usually glabrous. Their genome size estimates were more than 10 % lower than the mean genome size of all tetraploids, including all other Fair Isle samples (Fig. 3A). While these populations might have independently evolved lower genome sizes, it seems more plausible that they share variants underlying large differences in genome size such as missing dispensable chromosomes or chromosome regions, although these have yet to be reported (see Methods). An explanation of genomic homogenization in sympatry is in keeping with the growing body of plant research showing gene flow at the early stages of species divergence, or between closely related species (e.g. Strasburg & Rieseberg, 2008; Papadopulos *et al.*, 2011; Brandvain *et al.*, 2014; Sawangproh *et al.*, 2020). Such observations of divergence with gene flow are often coupled with species differences being maintained by a few diverged regions under strong selection maintaining species identities (e.g. Twyford & Friedman, 2015), a possibility we are currently investigating in *Euphrasia*.

Within three of the widespread species that we sampled extensively, we found considerably higher genome size variation in the mainly outcrossing *E. anglica* and *E. arctica* than in highly selfing *E. micrantha*. Unlike the outcrossing species, *E. micrantha* shows no increase in genome size at higher latitudes, and instead the genome size is consistent across the species range. Lower diversity is expected in young selfing lineages such as *E. micrantha* for several reasons. First, selfing reduces the effective population size, resulting in lower genetic variation (Nordborg, 1997), presumably including presence/absence variants. Second, the reduced effective rate of crossing over between the chromosomes of a selfing species further reduces the effective population size (Conway *et al.*, 1999). Third, selfing species are rarely polymorphic for B chromosomes (Burt and Trivers, 2008), one source of genome size variation in the Orobanchaceae, for instance in closely related *Rhinanthus* (Wulff, 1939; Hambler, 1953). Finally, partially selfing species are less likely to acquire genome size variants through introgression (e.g. Pajkovic *et al.*, 2014). Older highly selfing lineages may, however, have diversified ecologically and become restricted to different habitats, and might evolve genome size differences.

### Genome size differences and genomic repeats

We found very low differentiation of genomic repeats between species of British *Euphrasia*, and our analysis of the most abundant clusters failed to detect any species-specific repeats. Consistent with previous phylogenetic work on British *Euphrasia* (Wang *et al.*, 2018), there were no examples where all individuals of a given species cluster together based on repeat content (Fig. 4A). The fact that species of British *Euphrasia* are closely related and often hybridize makes lineage-specific large-scale gains or losses of individual repeat groups, as seen in other plants (Piegu *et al.*, 2006; Macas *et al.*, 2015; McCann *et al.*, 2020), an unlikely cause for genome size variation in *Euphrasia*. Instead, the observed differences are probably due to changes in numerous different repeats or low-copy sequences segregating within the *Euphrasia* gene pool. At present, it is hard to tell whether these presence/absence variants comprise numerous individual repeat copies or whether there are (also) larger-scale presence/absence variants such as the loss or gain of chromosome fragments, as hypothesized to be present in hybridizing species of *Anacyclus* (Agudo *et al.*, 2019; Vitales *et al.*, 2020). The high frequency of hybridization in *Euphrasia* may lead to increased levels of structural rearrangements due to ectopic recombination, which may be more common between heterozygous genomic repeats (Morgan, 2001).

Between ploidy levels of British *Euphrasia*, we found that the closely related allotetraploids had an 11 % lower mean genome size compared with the value predicted from doubling the mean genome size of the closely related diploids. This discrepancy may have originated from genome downsizing following polyploidy as commonly seen during re-diploidization. It may also have resulted from the fusion of two diploid progenitor genomes of different size, as seen in allopolyploid *Gossypium* (Hendrix and Stewart, 2005) and *Arabidopsis suecica* (Burns *et al.*, 2021). Finally, the genome sizes of diploids and tetraploids may have evolved in different directions after the formation of the tetraploids. The absence of clear interploidy repeat divergence in *Euphrasia* differs from other allotetraploid systems, where diverged sub-genomes tend to show large-scale differences in genomic repeats (Zhao *et al.*, 1998; Hawkins *et al.*, 2006; Renny-Byfield *et al.*, 2015; Dodsworth *et al.*, 2020). However, there was some ploidy-associated variation in several tandem repeat clusters, possibly indicating sub-genome-specific satellite differences in the allopolyploids, as observed in *Chenopodium quinoa* (Heitkam *et al.*, 2020). The lack of larger-scale repeat differentiation between diploids and tetraploids is notable because nuclear k-mer spectra (Becher *et al.*, 2020) and rDNA sequences (X. Wang *et al.*, 2018) suggest considerable sequence divergence between the tetraploid sub-genomes, corresponding to a split of ~8 Myr (Gussarova *et al.*, 2008).

Tandem repeats such as rDNA and other satellite DNAs are generally found to be the fastest evolving fraction of the repeatome, showing divergence in both copy number and sequence between closely related species (e.g. Tek *et al.*, 2005; Ambrozová *et al.*, 2011; Renny-Byfield *et al.*, 2012; Becher *et al.*, 2014; Ávila Robledillo *et al.*, 2020) and populations (Ananiev *et al.*, 1998). We confirmed this in *Euphrasia*, where tandem repeats accounted for the eight repeat clusters with the highest inter-individual variation in genomic abundance (Fig. 4D). While differing across individuals, repeat content did not show any clear signal of divergence between species. For example, there was no obvious signal of divergence in a comparison between *E. micrantha* and divergent tetraploids such as *E. arctica*. This is surprising not just because of their morphological distinctiveness, but also their difference in outcrossing rate, with theory predicting that the copy-number and equilibrium frequency of transposable elements depends on the level of selfing in a population (Morgan, 2001; Dolgin and Charlesworth, 2006). A probable explanation is that the shift to high-selfing in *E. micrantha* is relatively recent compared to the time it takes for the genomic repeat content to reach equilibrium level.

### Evolution of genome size variation

The continuous genome size variation within and between *Euphrasia* species, coupled with these differences probably being a product of segregating presence/absence variants across the genome, underlines the polygenic nature of genome size variation. Regarding genome size differences to be the result of segregating genetic variants blurs the classic distinction between genotype and nucleotype, where ‘nucleotype’ refers to ‘conditions of the nucleus that affect the phenotype independently of the informational content of the DNA’, a definition essentially identical to genome size (Bennett, 1971, 1977). Because genome size has been shown to be correlated with many traits including cell size, stomatal pore size, the duration of cell division and life-history differences (e.g. Šímová & Herben, 2012; Bilinski *et al.*, 2018; Roddy *et al.*, 2020), it is plausible that it is affected indirectly by selection on such traits. There might be additional indirect selection on genome size according to the mutational-hazard hypothesis (e.g. Lynch, 2011), which proposes that a large genome size may be selected against because there is more opportunity for the accumulation of deleterious mutations.

It follows that individual presence/absence variants may be under different kinds of simultaneous selection, potentially of different directionality. For instance, there might be positive selection on an adaptive insertion, which is simultaneously selected against because it increases genome size. Further, because selection at one locus affects regions that are physically linked (i.e. selection at linked sites, Maynard Smith & Haigh, 1974; Charlesworth *et al.*, 1993), the footprint of selection on genome regions is modified by the (effective) rate of crossing over, which varies along genomes and between mating systems.

Research on genome size is somewhat decoupled from studies on sequence-based variation in populations. We suggest future research into genome size evolution should consider both patterns of total genome size and the population processes underlying this variation. In addition to furthering our understanding of intraspecific genome size diversity in *Euphrasia* and other plant groups, answers to these questions will also improve our understanding of genome size evolution, which starts at the individual and population level.

## SUPPLEMENTARY DATA

Supplementary data are available online at https://academic.oup.com/aob and consist of the following. Figure S1. Flow cytometry histograms. A diploid and a tetraploid sample. Intraspecific GS variation in a diploid and a tetraploid species. Figure S2. Genome size plotted against latitude. Figure S3. The relative abundance of the 100 largest repeat clusters in 30 samples of *Euphrasia.* Table S1. Genome size estimates and sample data. Table S2. Details of the whole-genome sequencing datasets generated and genomic proportions of repeat types.

mcab102_suppl_Supplementary_Figure_S1

mcab102_suppl_Supplementary_Figure_S2

mcab102_suppl_Supplementary_Figure_S3

mcab102_suppl_Supplementary_Table_S1

mcab102_suppl_Supplementary_Table_S2
