# Supplementary material for: The nature of intraspecific and interspecific genome size variation in taxonomically complex eyebrights
Source: Ann Bot. 2021 Jul 28;128(5):639–51. doi: 10.1093/aob/mcab102 (PMC8422891; doi:10.1093/aob/mcab102)
Supplement: mcab102_suppl_Supplementary_Figure_S1 [file mcab102_suppl_supplementary_figure_s1.pdf]

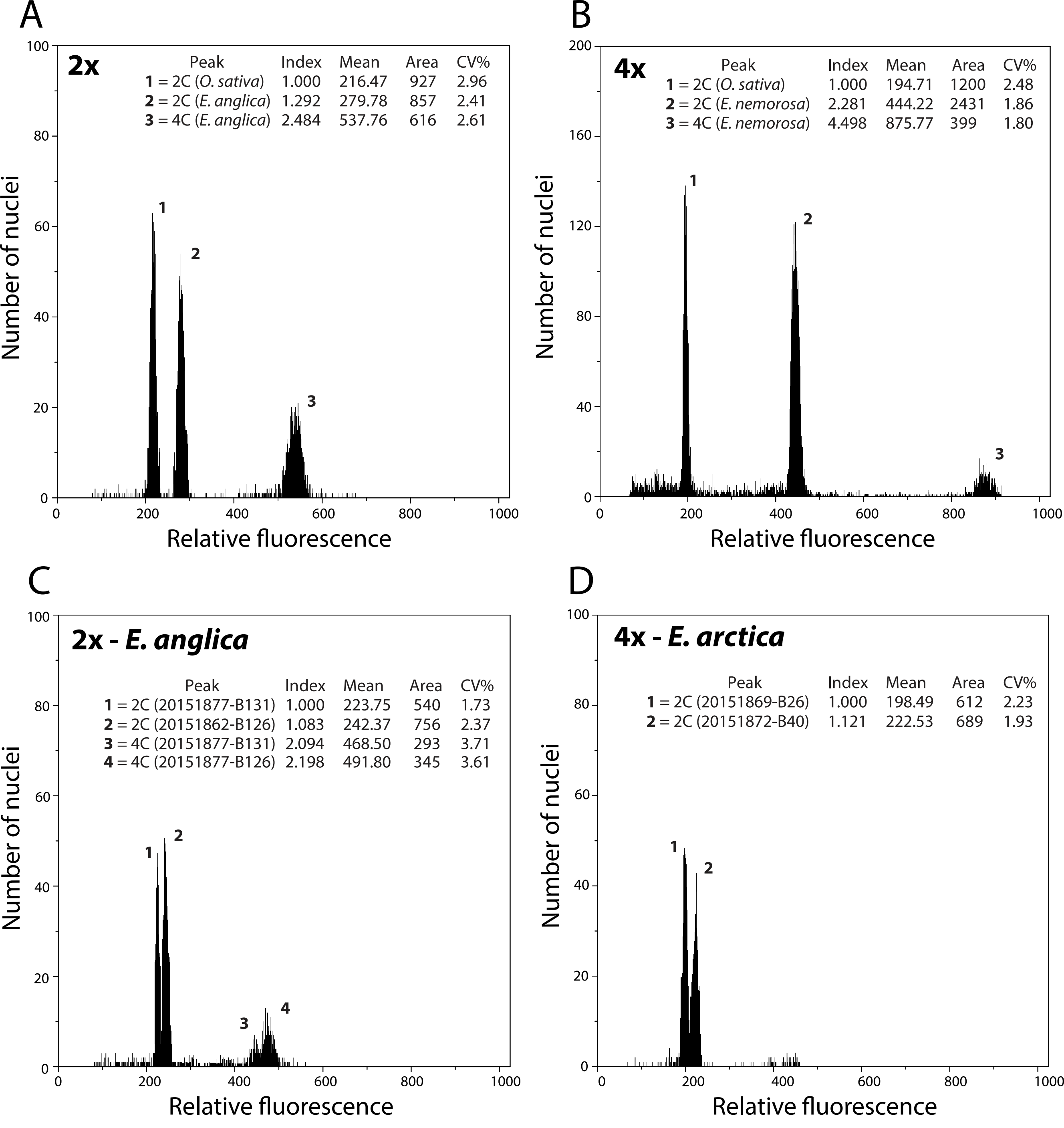


Supplementary data Figure S1. Flow cytometry histograms. A diploid (A) and a tetraploid (B) sample. Intraspecific GS variation in a diploid (C) and a tetraploid (D) species.
