# Supplementary figures and images for: The nature of intraspecific and interspecific genome size variation in taxonomically complex eyebrights
Source: Ann Bot. 2021 Jul 28;128(5):639–51. doi: 10.1093/aob/mcab102 (PMC8422891; doi:10.1093/aob/mcab102)

# GS by latitude

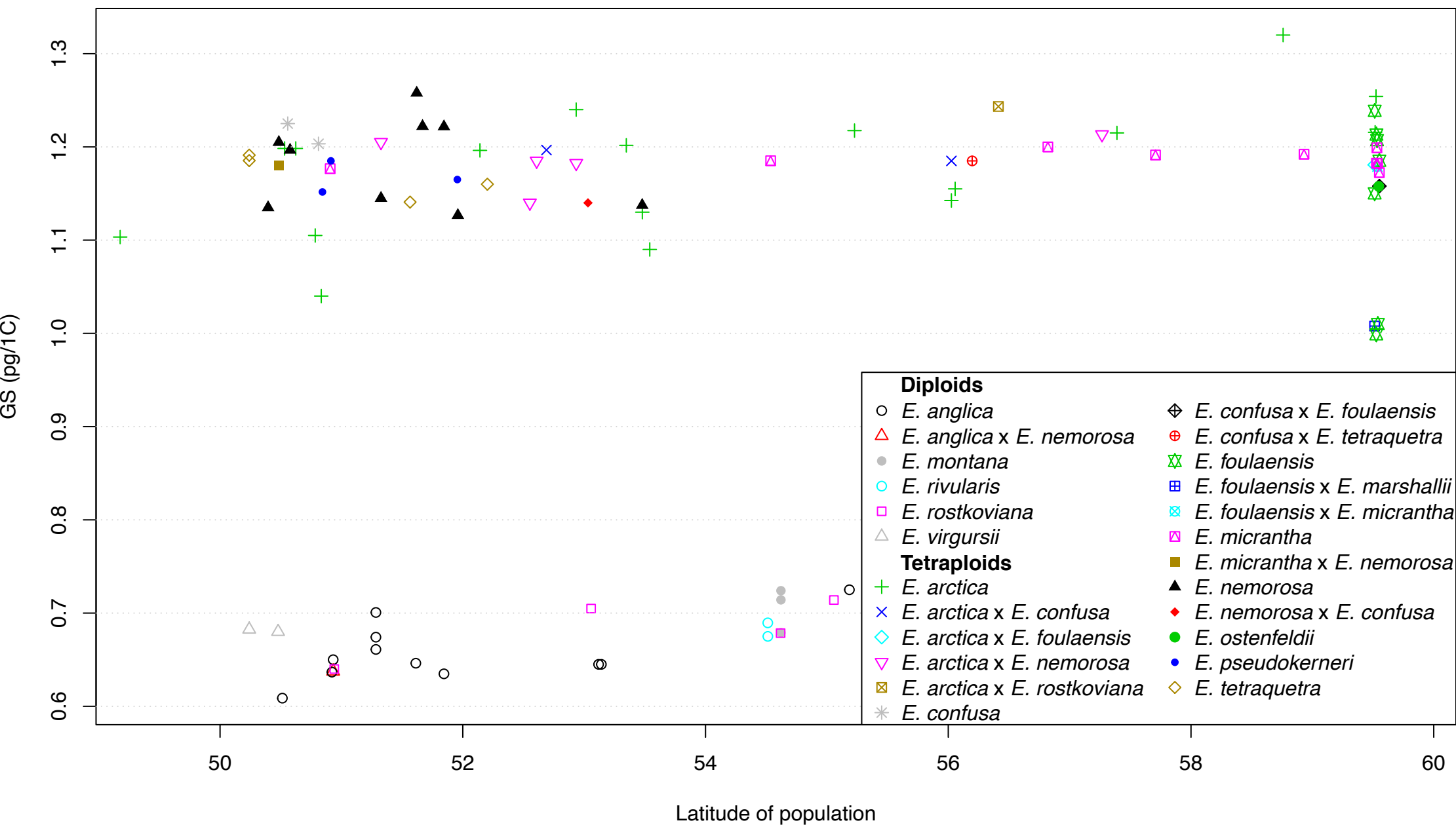

Supplementary data Figure S2. Genome size plotted against latitude.

Supplement: mcab102_suppl_Supplementary_Figure_S2 [file mcab102_suppl_supplementary_figure_s2.pdf]
