# Supplementary material for: The nature of intraspecific and interspecific genome size variation in taxonomically complex eyebrights
Source: Ann Bot. 2021 Jul 28;128(5):639–51. doi: 10.1093/aob/mcab102 (PMC8422891; doi:10.1093/aob/mcab102)
Supplement: mcab102_suppl_Supplementary_Figure_S3 [file mcab102_suppl_supplementary_figure_s3.pdf]

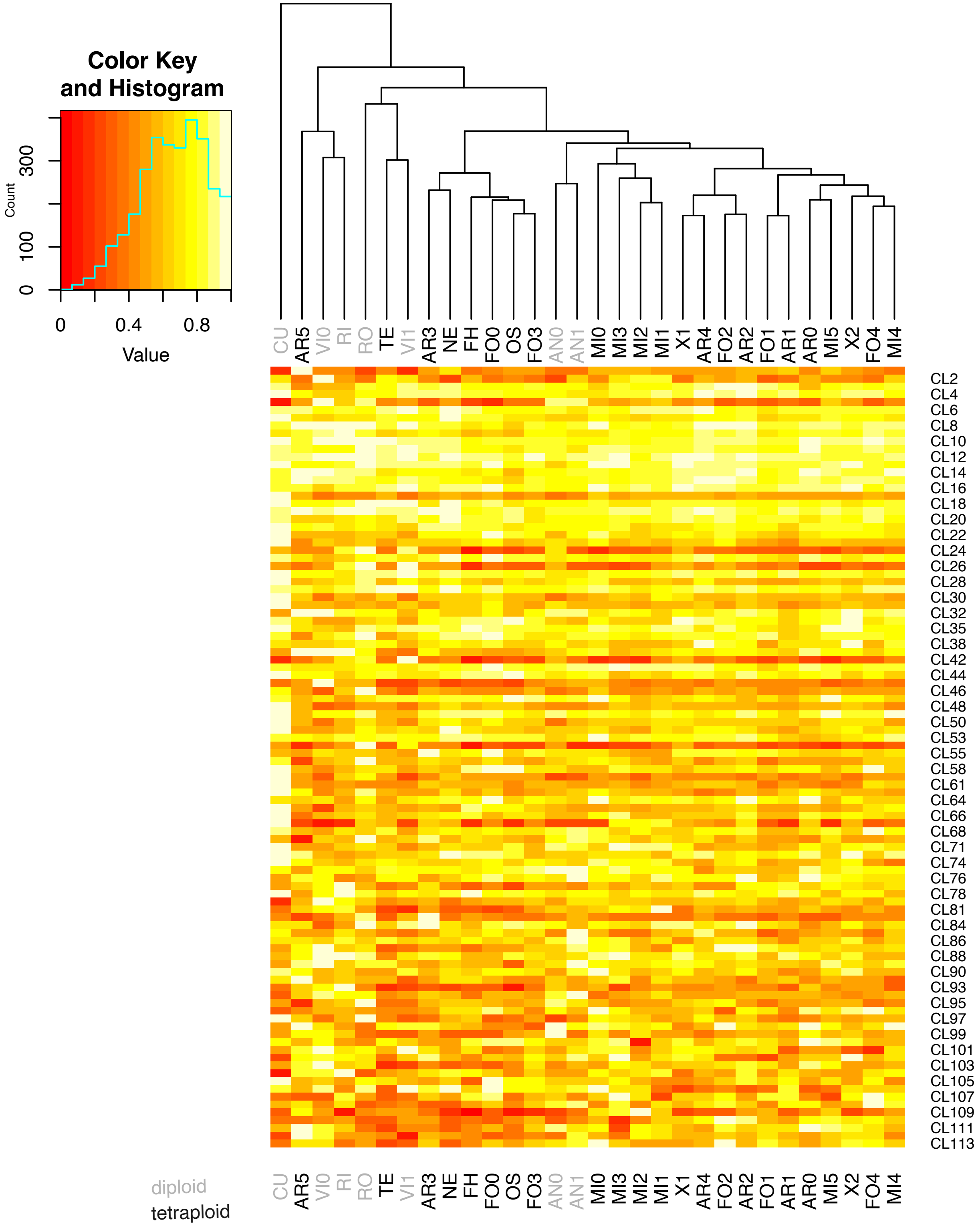

Supplementary data Figure S3. The relative abundance of the 100 largest repeat clusters in 30 samples of *Euphrasia* (excluding plastid sequences and clusters private to *Bartsia alpina*). For each cluster, the read counts were scaled proportionally across samples, setting the largest read count to 1 (yellow-white).
